# Decoding molnupiravir-induced mutagenesis in SARS-CoV-2

**DOI:** 10.1016/j.jbc.2021.100867

**Published:** 2021-06-09

**Authors:** Luis Menéndez-Arias

**Affiliations:** Centro de Biología Molecular “Severo Ochoa”, Consejo Superior de Investigaciones Científicas & Universidad Autónoma de Madrid, Madrid, Spain

**Keywords:** molnupiravir, SARS-CoV-2, COVID-19, RNA polymerase, lethal mutagenesis, antiviral drug, COVID-19, coronavirus infectious disease 2019, MERS-CoV, Middle East respiratory syndrome coronavirus, NHC, β-D-*N*^4^-hydroxycytidine, RdRp, RNA-dependent RNA polymerase, rNTP, ribonucleoside triphosphate, SARS-CoV, severe acute respiratory syndrome coronavirus

## Abstract

Molnupiravir, a prodrug of the nucleoside derivative β-D-*N*^4^-hydroxycytidine (NHC), is currently in clinical trials for COVID-19 therapy. However, the biochemical mechanisms involved in molnupiravir-induced mutagenesis had not been explored. In a recent study, Gordon *et al.* demonstrated that NHC can be incorporated into viral RNA and subsequently extended and used as template for RNA-dependent RNA synthesis, proposing a mutagenesis model consistent with available virological evidence. Their study uncovers molecular mechanisms by which molnupiravir drives SARS-CoV-2 into error catastrophe.

Outbreaks of highly pathogenic severe acute respiratory syndrome coronavirus (SARS-CoV) in 2002 to 2003 and Middle East respiratory syndrome coronavirus (MERS-CoV) in 2012 raised the alarm on the potential threat of coronaviruses to human health. Today, we are witnessing a huge pandemic caused by SARS-CoV-2 that has infected millions of people, while causing more than 3.7 million deaths around the world. Although a handful of vaccines are now available, there is a lack of effective antiviral drugs to fight the disease caused by SARS-CoV-2 infection (COVID-19).

Coronaviruses are enveloped viruses with a positive-sense single-stranded RNA genome of 26.4 to 31.7 kilobases (the largest of all RNA virus genomes). The coronavirus genome encodes nonstructural proteins that are responsible for the replication and transcription of the viral genome, the main component of which is a multifunctional protein that contains a central RNA-dependent RNA polymerase (RdRp) domain (1). Viral polymerases are common targets of antiviral intervention, and nucleoside and nucleotide analogues constitute the backbone of the most effective antiviral therapies (2). A nucleotide analogue known as remdesivir remains as the only drug approved by regulatory agencies to treat COVID-19. However, some clinical studies have failed to confirm its beneficial effects. In addition, the drug is difficult to synthesize, expensive, and has to be administered intravenously in a hospital setting. These attributes are undesirable in the context of a pandemic and efforts have been focused on the development of alternative inhibitors of SARS-CoV-2 replication. Among them, molnupiravir has emerged as a promising new drug targeting the coronavirus RNA polymerase.

Molnupiravir is the isopropylester prodrug of the ribonucleoside analogue β-D-*N*^4^-hydroxycytidine (NHC). NHC is a broad-spectrum antiviral compound that inhibits the replication of multiple viruses in cell culture (*e.g.*, Chikungunya virus, Venezuela equine encephalitis virus, respiratory syncytial virus, hepatitis C virus, norovirus, influenza A and B viruses, Ebola virus, and human coronaviruses) (3). The triphosphorylated derivative of NHC is a substrate for viral RNA polymerases and interferes with viral replication. In cell culture assays, molnupiravir was found to be a potent inhibitor of SARS-CoV-2 replication with an EC_50_ in the submicromolar range (3, 4). This inhibitory effect was also observed in animal models such as Syrian hamsters and humanized mice (5, 6), and NHC administration to infected ferrets prevented SARS-CoV-2 transmission to untreated and uninfected animals (7). Molnupiravir is currently in phase II/III clinical trials based on encouraging preclinical data and its lack of toxicity and adverse side effects in phase I clinical trials. Studies carried out in cell culture with alphaviruses and coronaviruses have shown the mutagenic action of NHC, inducing G to A and C to U transitions in a dose-dependent manner. However, the biochemical events mediating these effects had not been studied at a biochemical level.

In their recent work, Gordon *et al.* (8) used a SARS-CoV-2 RdRp holoenzyme and various RNA-RNA template primers to determine nucleotide incorporation efficiencies for NHC triphosphate and natural ribonucleoside triphosphates (rNTPs) in different sequence contexts. Although accurate determinations of *k*_pol_ (polymerase catalytic rate constant) and *K*_d_ (nucleotide binding affinity) were not obtained, their results clearly showed that all rNTPs are more efficiently incorporated than the NHC triphosphate. However, as expected, the most efficient competition was observed with cytosine triphosphate (CTP). In a different set of experiments, Gordon *et al.* found that once incorporated, primers having NHC monophosphate at their 3’ end were efficiently extended, particularly at high rNTP concentrations.

This observation led the authors to investigate whether a template NHC could have an impact on the generation of errors during SARS-CoV-2 RNA synthesis. An RNA template containing an embedded NHC monophosphate was synthesized and used in RNA elongation experiments, which demonstrated the formation of both NHC:A and NHC:G base pairs. However, efficient extension was only observed in the case of NHC:A base pairing. These biochemical data led the authors to propose a model to explain the mutagenic antiviral action of NHC (Fig. 1). While the formation of NHC:G base pairs could lead to RNA synthesis inhibition, NHC:A pairing induced mutagenesis by increasing G to A and C to U transition frequencies, in agreement with evidence collected from previous experiments carried out *in vitro* and *in vivo* (3, 6). The RdRp holoenzyme used in the experiments lacked the exoribonuclease protein found in the SARS-CoV-2 replicase-transcriptase complex; however, the proofreading activity of the related MERS-CoV protein is effectively inhibited by NHC (3), and therefore, the conclusions drawn on molnupiravir action for the RNA polymerase holoenzyme are expected to be valid for the full replicase-transcriptase complex.

**Figure 1:**
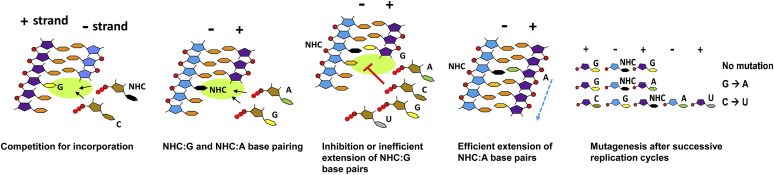
**Proposed model for the antiviral mechanism of molnupiravir, based on NHC-induced mutagenesis and inhibition of RNA synthesis**.

Lethal mutagenesis is a broad-spectrum antiviral strategy that exploits the high mutation rate and low mutational tolerance of many RNA viruses. A number of successful viral extinctions in cell culture using other mutagenic nucleosides have been reported. However, lethal mutagenesis still needs validation in animals and humans. A mutagenic effect has been shown in different viruses for approved nucleosides such as ribavirin and favipiravir. While ribavirin increases G to A and C to U mutation frequencies, favipiravir facilitates A to G and U to C transitions (reviewed in ref. (9)). Approved in Japan for the treatment of influenza, favipiravir is also licensed in China and Russia for COVID-19 treatment. A recent study by Zhou *et al.* (10) showed that NHC is more than 100-fold more active than ribavirin and favipiravir against SARS-CoV-2, while the observed activity correlated with an increased mutation frequency in the viral RNA. It would be interesting to see whether the methodological approach of Gordon *et al.* (8) could throw some light on the potential mechanism of action of favipiravir in the context of SARS-CoV-2 and influenza virus infections.

Available evidence suggests that molnupiravir could become a paradigmatic example in the use of lethal mutagenesis as an antiviral strategy. However, there are inherent risks in this approach. NHC can be metabolized by the host cell to the 2′-deoxyribonucleoside form by the ribonucleotide reductase and then incorporated into the host cell DNA. The mutagenic effect of NHC has been shown in animal cell cultures (10), raising concerns on the potential risk of molnupiravir-induced tumorigenesis and the emergence of detrimental mutations in sperm precursor cell generation and embryo development.

## Conflict of interest

The author declares that he has no conflicts of interest with the contents of this article.
